# Changes in chronic disease prevention resources and activities in Canada during the COVID-19 pandemic

**DOI:** 10.24095/hpcdp.45.7/8.03

**Published:** 2025-08

**Authors:** Katerina Maximova, Maryam Marashi, Elizabeth Holmes, David L. Mowat, Greg Penney, Gilles Paradis, Jennifer L. O’Loughlin

**Affiliations:** 1 MAP Centre for Urban Health Solutions, Li Ka Shing Knowledge Institute, St. Michael’s Hospital, Toronto, Ontario, Canada; 2 Dalla Lana School of Public Health, University of Toronto, Toronto, Ontario, Canada; 3 Faculty of Kinesiology and Physical Education, University of Toronto, Toronto, Ontario, Canada; 4 Canadian Cancer Society, Toronto, Ontario, Canada; 5 Canadian Partnership Against Cancer, Toronto, Ontario, Canada; 6 Canadian Public Health Association, Ottawa, Ontario, Canada; 7 Department of Epidemiology, Biostatistics and Occupational Health, McGill University, Montral, Quebec, Canada; 8 Institut national de sant publique du Qubec, Montral, Quebec, Canada; 9 Centre de recherche du centre hospitalier de l’Universit de Montral (CRCHUM), Montral, Quebec, Canada; 10 cole de sant publique de l’Universit de Montral, Montral, Quebec, Canada

**Keywords:** chronic disease prevention, resources, activities, Canada, COVID-19, pandemic, survey, noncommunicable disease, NCD

## Abstract

**Background:**

The COVID-19 pandemic disrupted public health efforts for chronic disease prevention (CDP) in Canada and elsewhere. We describe COVID-19–related disruptions in CDP resources and activities among Canadian public health organizations.

**Methods:**

We surveyed all organizations in Canada with mandates for primary CDP, including “resource organizations” that develop or transfer CDP initiatives and “user organizations” that deliver these CDP initiatives to target populations. Key informants most knowledgeable about CDP activities and resources within each organization reported pandemic-related changes in CDP resources and activities. User organizations also reported on the status of 18 specific CDP activities and rated whether pandemic containment measures were barriers to or facilitators of CDP activities.

**Results:**

Of the 298 participating organizations (88% response), 129 were resource organizations (37% formally mandated organizations [FMOs]; 63% non-governmental organizations [NGOs]) and 169 were user organizations (48% FMOs; 52% NGOs). Overall, 36% reported decreases in CDP funding (24% major, 12% minor), 30%–41% reported decreases in full-time, volunteer and managerial staff (19%–27% major, 11%–14% minor) and 32% reported decreases in CDP activities (23% major, 9% minor). User FMOs were most affected by decreases. Among user organizations, 16%–39% decreased, suspended or discontinued specific CDP activities. Still, 8%–39% increased their activities, particularly those targeting mental health, marginalized populations, racialized communities and specific gender groups. Half (53%) of user organizations perceived COVID-19 contagion restrictions as barriers to CDP activities.

**Conclusion:**

Continued monitoring of CDP resources and activities can inform emergency preparedness and ensure that CDP remains a priority during public health crises.

**Highlights**

- At least one-third (30%–41%) of public health organizations reported decreases in chronic disease prevention (CDP) funding, personnel and activities during the COVID-19 pandemic.
- Formally mandated public health user organizations had particularly high decreases in CDP resources and activities.
- There were marked decreases in tobacco control, healthy eating and healthy weight activities.
- Activities targeting mental health, marginalized populations, racialized communities and specific gender groups increased.
- More than half of user organizations viewed COVID-19 public health measures as barriers to CDP activities.

## Introduction

The COVID-19 pandemic placed unprecedented strain on Canada’s health care and public health systems and radically affected delivery of prevention programs and services for chronic diseases. According to a World Health Organization survey, 75% of countries reported disruptions in chronic disease services, including hypertension management, diabetes management and cancer treatment.^1^ Other studies reported notable declines in cancer screening test volumes, including in Canada, at the start of the pandemic,^2^,^3^ leading to delayed diagnoses and treatments.

Although less apparent or documented, the COVID-19 pandemic also disrupted preventive efforts in the public health system. In Canada and elsewhere, public health system capacity (i.e. skills and resources) dedicated to chronic disease prevention (CDP) was diverted to combat the spread of COVID-19. Globally, 20% of countries reported reassignment and deployment of full-time CDP staff to support COVID-19 efforts, leading to reductions in the capacity of public health systems to undertake usual CDP activities.^1^

The development and delivery of programs, policies and practices with the aim of preventing chronic (or noncommunicable) diseases such as cancer, cardiovascular disease, diabetes, chronic respiratory illness, mental illness are critical components of Canada’s public health systems.^4^-^6^ Public health systems and, more specifically, organizations within public health systems with mandates for CDP are vital to reducing the chronic disease burden, but are chronically underfunded and underprioritized and frequently undergo restructuring and reform.^7^-^10^

CDP organizations have diverse mandates, missions, structures, target populations and funding. This research team previously characterized CDP organizations as either “resource organizations,” which develop CDP programs, policies and practices and then transfer these initiatives to other organizations, or “user organizations,” which deliver CDP initiatives to the general population or to specific population groups.^11^,^12^ These organizations can vary from formally mandated organizations (FMOs) to non-governmental or nonprofit organizations (NGOs). FMOs are governmental and arms-length governmental organizations with a formally mandated, legislated role in CDP, for example, health authorities and public health units. NGOs include non-governmental, nonprofit organizations, health charities, professional associations, research centres and resource centres.^13^ While FMOs generally have more stable resources, including funding and personnel, NGOs rely heavily on volunteer support and report more challenges with adequate funding and stability.^11^

Improved understanding of the extent to which the COVID-19 pandemic disrupted the functioning of public health organizations engaged in CDP would help to build (or rebuild) resilient public health systems capable of withstanding future health crises and natural disasters. Further, to ensure a coordinated response to CDP, we must understand whether different types of organizations (resource versus user, FMO versus NGO) were impacted differently. No studies to date have investigated changes in public health organizations’ CDP resources (funding or personnel) or activities (programs, policies or practices) during the COVID-19 pandemic at the national, provincial or regional levels in Canada. In this study, we sought to describe (1) changes in CDP resources (funding and personnel) and activities (programs, policies and practices) in resource and user FMOs and NGOs; (2)changes in 18 specific CDP activities targeting lifestyle risk factors, chronic disease diagnoses, mental health and specific population groups in user FMOs and NGOs; and (3) whether user FMOs and NGOs perceived public health measures to curtail the spread of COVID-19 as barriers or facilitators to CDP activities.

## Methods

***Ethics approval***

The study was approved by the Ethics Review Boards at St. Michael’s Hospital, Unity Health Toronto (REB #21-240) and Centre hospitalier de l’Universit de Montral (CRCHUM) (F9H-86805).

***Study population***

The Public Health ORganizational CApacity STudy (PHORCAST) is a repeat national census of all public health organizations in Canada engaged in CDP at the national, provincial or territorial, or regional levels. The organizations in PHORCAST have been characterized as resource and user organizations.^11^,^12^ Organizations that have both resource and user mandates or functions are considered as unique, separate entities.

Data were collected in 2004, 2010 and 2023 from all resource and user organizations with mandates for population-level CDP identified through extensive online searches and consultation with key informants with wide-ranging knowledge of the public health landscape in Canada.^11^-^13^ This current study uses data drawn from the 2023 data collection cycle.

New organizations identified in 2023 included those that were established after the 2010 data collection wave; pre-existing organizations with new CDP activities or with newly formed CDP divisions; and organizations formed through the amalgamation of two or more previously participating organizations. Excluded were local-level organizations; grouped organizations (i.e. coalitions, partnerships, alliances); organizations primarily engaged in secondary or tertiary prevention, advocacy, allocation of funds, fundraising and facilitating joint interorganizational efforts; and organizations exclusively engaged in research or knowledge transfer.

***Procedures***

All resource and user organizations identified in 2023 (n = 321) were screened for eligibility. We sent an introductory email to a senior manager in each organization to solicit participation, confirm eligibility and establish whether the organization was a resource or user organization or both. The senior manager was then asked to identify a key informant for data collection, that is, the individual who was most knowledgeable about CDP activities and resources within the organization. Senior managers could suggest themselves as the key informant. We contacted key informants via email to introduce the study, notify them of their senior manager’s consent and confirm their suitability as the key informant; we followed up with nonrespondents through repeat emails and telephone calls.

Key informants were emailed a copy of the relevant questionnaire (to share with colleagues if they needed help with responses to any questions) and a personalized link to the 45- to 60-minute-long English or French questionnaire, available online on the LimeSurvey platform (LimeSurvey GmbH, Hamburg, DE). To facilitate survey completion and accommodate their schedules, the key informants (henceforth referred to as participants) could complete the questionnaire in an interview with the study coordinator or investigators over Zoom (Zoom Communications, San Jose, CA, US) or by telephone. After completing the questionnaire, the key informants were asked for any open-ended comments.

***Measures***

**Changes in CDP funding, personnel and activities during the COVID-19 pandemic**

Participants in both resource and user organizations reported the extent of perceived changes (major decreases, minor decreases, no change, minor increases, major increases) in funds spent on CDP during the COVID-19 pandemic; the number of full-time employees, volunteers (including Board members) and managers involved in CDP; and CDP activities (programs, policies and practices).

**Changes in 18 specific CDP activities during the COVID-19 pandemic**

Participants in user organizations only (i.e. those organizations that deliver CDP activities to populations) were asked to report any changes in 18 CDP activities during the COVID-19 pandemic: lifestyle risk factors, including tobacco control, healthy eating, physical activity, healthy lifestyle, and prevention of high blood pressure and of high cholesterol; chronic disease diagnoses, including chronic obstructive pulmonary disease (COPD), diabetes, cancer, heart disease, healthy weights; stress and mental health; and marginalized populations, racialized groups or communities and specific gender groups (i.e. women, men or gender-diverse people) as well as rural communities and urban communities. Specifically, participants were asked whether each of these CDP activities had changed in the past 3 years and, if so, whether these changes occurred before or during the COVID-19 pandemic. We determined whether each of the 18 activities remained stable, had increased, had decreased, was temporarily suspended or was permanently discontinued during the COVID-19 pandemic.

**The COVID-19 pandemic as a barrier or facilitator to CDP activities**

The participants in user organizations reported the extent to which public health measures to restrict COVID-19 contagion were barriers or facilitators to organizational CDP activities. Responses were recorded on a seven-point Likert scale with the following labels: “very strong barrier,” “strong barrier,” “somewhat strong barrier,” “neither barrier nor facilitator,” “very strong facilitator,” “strong facilitator” and “somewhat strong facilitator.”

**Organization type**

Organizations were categorized as FMOs or NGOs. FMOs include federal, provincial or territorial government departments; regional, provincial or territorial administrative health authorities; public health agencies and units; and para-governmental health organizations (i.e. arms-length organizations funded by the government but acting independently). NGOs include non-governmental, nonprofit organizations, health charities, professional associations, research centres and resource centres.

***Open-ended question***

Upon completing the questionnaire, participants could provide any other comments.

Detailed descriptions of study variables, including questionnaire item(s) and response choices, are provided in Supplemental Tables 1 and 2. These tables and other information, including recoding of responses for analysis, and the number and percentage of participants with missing data for each study variable, are available on request from the authors.

**Table 1:** Characteristics of resource and user FMOs and NGOs engaged in CDP, PHORCAST, Canada, 2023

Characteristics	Total (n = 298)	User organization (n = 169)		Resource organization (n = 129)	
		FMO (n = 81)	NGO (n = 88)	FMO (n = 48)	NGO (n = 81)
**Median age of organization (IQR), years **	49 (22–75)	50 (22–76)	50 (29–86)	40 (18–75)	39 (19–60)
Geographic area served, %					
Subregion	8	17	7	6	3
Region	28	48	19	27	19
Province/territory	44	32	48	58	44
Multiple provinces/territories	4	1	6	0	9
Canada	15	1	21	8	26
Population size, %					
< 50 000	8	10	8	4	10
50 000–99 999	3	4	2	4	1
100 000–199 999	16	25	13	15	12
200 000–499 999	14	22	10	10	12
500 000–1 000 000	13	10	16	13	14
> 1 000 000	46	30	51	54	51
**No. of full-time CDP staff, median (IQR) **	35 (9–200)	250 (130–3750)	15 (6–54)	200 (100–6000)	11 (6–30)
**No. of volunteers, median (IQR) **	20 (7–60)	11 (0–50)	35 (10–80)	0 (0–18)	23 (9–58)

**Abbreviations: **CDP, chronic disease prevention; FMO, formally mandated organization; IQR, interquartile range; NGO, nongovernmental organization; No., number. 

**Notes: **Resource organizations develop CDP programs, policies and practices and then transfer these initiatives to other organizations. User organizations deliver CDP programs, policies and practices to the general population or to underserved population groups. FMOs include federal, provincial or territorial government departments; regional, provincial or territorial administrative health authorities; public health agencies and units; and para-governmental health organizations (i.e. armslength organizations funded by the government but acting independently). NGOs include non-governmental, nonprofit organizations, health charities, professional associations, research centres and resource centres. 

**Table 2:** Percentage of resource and user organizations with CDP mandates, by reported change in CDP funding, personnel and activities during the COVID-19 pandemic, PHORCAST, Canada, 2023 (n = 298)

Area of change	Reported change				
	Major decreases, %	Minor decreases, %	No change, %	Minor increases, %	Major increases, %
Funds spent on CDP	24	12	39	19	6
No. of full-time staff involved in CDP	27	14	39	14	6
No. of volunteers involved in CDP	19	12	63	5	1
No. of managers involved in CDP	19	11	58	9	3
No. of CDP activities	23	9	32	22	8

**Abbreviations: **CDP, chronic disease prevention; No., number. 

**Note: **The sum of percentages is calculated across rows, for each area of change. 

***Data analyses***

Descriptive statistics were used to characterize organizations and report changes in CDP funding, personnel and activities during the COVID-19 pandemic. We computed the proportion of all CDP organizations reporting major decreases, minor decreases, no change, minor increases and major increases in CDP funding, personnel and activities. We then stratified resource and user organizations according to FMO or NGO status and reported these proportions in each of the resulting four groups. Organizations that were both resource and user organizations were considered separately as unique entities in these analyses (i.e. once in the user group and once in the resource group).

Next, we computed the proportions of user organizations reporting that delivery of each of 18 specific CDP activities had remained stable, had increased, had decreased, was temporarily suspended or was permanently discontinued during the COVID-19 pandemic. We used as the denominator the total number of organizations that reported undertaking the specific CDP activity in the last 3 years.

Finally, we computed the proportion of user organizations that selected each of the ratings on the seven-point Likert scale (from “very strong barrier” to “very strong facilitator”) describing how the COVID-19 contagion measures may have affected CDP activities.

Statistical significance was not relevant in these descriptive analyses of the census of CDP organizations in Canada.

Analyses were conducted using Stata version 17 (Stata Corp, College Station, TX, US). No formal qualitative analysis of these free-text responses was conducted. Excerpts from participants’ open-ended comments are included in this article to provide context and illustrate quantitative findings. Quotes are reproduced verbatim.

## Results

In 2023, PHORCAST surveyed 298 public health organizations with CDP mandates, which represented 88% of the eligible organizations. Of the 129 resource organizations, 37% were FMOs and 63% were NGOs. Of the 169 user organizations, 48% were FMOs and 52% were NGOs. The median organizational age was 49 years. These organizations served subregions (8%), regions (28%) or provinces or territories (44%) or were national in scope (15%). More than half (59%) served geographical areas with populations of 500 000 or more people. The median number of full-time staff and volunteers was 35 and 20, respectively (Table 1).

***Changes in CDP resources and activities during the COVID-19 pandemic***

More than one-third (36%) of organizations reported decreases in CDP funding, with 24% reporting these as major (Table2). Between 30% and 41% of all public health organizations across Canada reported decreases in dedicated full-time, volunteer and managerial staff during the first 3 years of the COVID-19 pandemic, with between 19% and 27% of the decreases characterized as major. Most notable were the decreases in the number of full-time employees involved in CDP (41%), with 27% of organizations reporting these decreases as major. About one-third (32%) of organizations reported decreases in CDP activities, with 23% characterized as major. Of note, 25% of organizations reported increases in CDP funding, 20% reported increases in the number of full-time employees involved in CDP, and 30% reported increases in CDP activities. However, most of these increases were characterized as minor.

Decreases in CDP funding, personnel and activities were more prevalent among FMOs than NGOs, and most markedly among user FMOs (Table 3). Among resource organizations, 38% of FMOs reported decreases in CDP funding, 52% in the number of full-time employees involved in CDP and 41% in CDP activities. These proportions were even higher among user FMOs, with 60% reporting decreases in funds spent on CDP, 71% in the number of full-time employees involved in CDP, 58% in the number of managers involved in CDP and 71% in CDP activities. On the other hand, a higher proportion of NGOs than of FMOs reported increases in CDP funding, personnel and activities during the pandemic, with differences between user NGOs and user FMOs the most striking.

**Table 3:** Percentage of resource and user FMOs and NGOs with CDP mandates, by reported change in CDP resources and activities during the COVID-19 pandemic, PHORCAST, Canada, 2023

Resource organizations (n = 129)	Reported change					
	Major/minor decreases, % FMO (n = 48)	No change, % FMO (n = 48)	Major/minor increases, % FMO (n = 48)	Major/minor decreases, % NGO (n = 81)	No change, % NGO (n = 81)	Major/minor increases, % NGO (n = 81)
Funds spent on CDP	38	40	23	22	44	34
No. of full-time staff involved in CDP	52	27	21	22	53	24
No. of volunteers involved in CDP	19	79	2	27	64	9
No. of managers involved in CDP	29	58	13	13	68	18
No. of CDP activities	41	38	22	14	53	34

**Table 3B:** Percentage of resource and user FMOs and NGOs with CDP mandates, by reported change in CDP resources and activities during the COVID-19 pandemic, PHORCAST, Canada, 2023

User organizations (n = 169)	Reported change					
	Major/minor decreases, % FMO (n = 81)	No change, % FMO (n = 81)	Major/minor increases, % FMO (n = 81)	Major/minor decreases, % NGO (n = 88)	No change, % NGO (n = 88)	Major/minor increases, % NGO (n = 88)
Funds spent on CDP	60	27	14	27	46	27
No. of full-time staff involved in CDP	71	17	12	26	53	22
No. of volunteers involved in CDP	34	61	5	37	56	7
No. of managers involved in CDP	58	36	5	19	71	12
No. of CDP activities	71	11	18	14	36	50

**Abbreviations: **CDP, chronic disease prevention; FMO, formally mandated organization; NGO, non-governmental organization; No., number. 

**Note:** The sum of percentages is calculated across rows, for each area of change. 

A participant at an FMO made this insightful comment:

The vast majority of our health promotion staff were redeployed to the COVID-19 response during the pandemic. We needed every available person to work on case and contact management and left health promotion with a skeleton staff for over 18 months. Public health was swamped and did what was needed to survive. We had limited resources that were negligible during COVID. Prior to COVID we did not have resources for internal evaluation, but lacking that, we did have an incredibly knowledgeable and dedicated team who research best practice both in terms of intervention but also process. 

Compared to FMOs, markedly lower proportions of NGOs reported decreases in CDP funding, personnel and activities during the COVID-19 pandemic, with decreases in the number of volunteers most common among resource and user NGOs (27% and 37%, respectively). Less than one-third of resource and user NGOs reported decreases in CDP funding (22% and 27%, respectively), in full-time CDP staff (22% and 26%, respectively), in the number of managers (13% and 19%, respectively) and in CDP activities (14% each). Compared to FMOs, higher proportions of NGOs reported increases in CDP funding, personnel and activities during the pandemic. Notably, CDP activities in resource and user NGOs intensified (34% and 50%, respectively) as did CDP funding (34% and 27%, respectively).

***Changes in specific CDP activities during the COVID-19 pandemic***

Of the user organizations that delivered one or more of 18 specific CDP activities in the past 3 years, 16% to 39% reported that the activities had decreased, were temporarily suspended or were permanently discontinued during the COVID-19 pandemic (Table 4). Relatively high proportions of organizations with activities that targeted tobacco control (39%), healthy eating (35%), physical activity (33%) and healthy weights (37%) in the past 3 years reported decreases, suspensions or discontinuations. Only 16% of organizations that undertook activities targeting elevated cholesterol reported decreased, suspended or discontinued activities. Organizations reported increases in programming that targeted mental health (39%), stress (30%), marginalized populations (32%), racialized groups or communities (33%) and specific gender groups (32%).

**Table 4:** Percentage of user organizations that undertook specific CDP activities in the past 3 years, by status of each activity during the COVID-19 pandemic, PHORCAST, Canada, 2023 (n = 169)

Area	Organizations that offered activity in past 3 years, n	Status of activity during the COVID-19 pandemic			
		Suspended/ discontinued, %	Decreased, %	Remained stable, %	Increased, %
Lifestyle risk factors					
Tobacco control	99	21	18	48	13
Healthy eating	124	21	14	52	13
Physical activity	142	21	12	55	12
Healthy lifestyle	136	17	13	57	14
High blood pressure	38	16	8	58	18
Elevated cholesterol	25	8	8	64	20
Chronic disease diagnoses					
COPD	45	14	16	53	18
Diabetes	55	16	11	56	16
Cancer	54	13	11	48	28
Heart disease	47	15	13	64	8
Healthy weights	85	21	16	54	8
Mental health					
Stress	104	14	15	43	30
Mental health	134	11	14	38	39
Population groups					
Marginalized populations	142	8	17	43	32
Racialized groups/communities	118	9	16	42	33
Specific gender groups	94	10	14	45	32
Rural communities	132	10	18	54	18
Urban communities	120	9	19	56	16

**Abbreviations:** CDP, chronic disease prevention; COPD, chronic obstructive pulmonary disease.

A comment by a study participant at an NGO underscores the growing emphasis on broader social determinants of health as evidenced by the increases in programs specifically targeting marginalized and racialized populations as a driver for program change:

We focus on the community as a client, not individuals, and are focusing away from modifiable risk factors to equity, racial equity, built environment, etc. We are learning and growing and challenging ourselves with modest resources to try to create greatest positive health impact while challenging ourselves to better understand unintended consequences and to be humble and open to two-eyed seeing and new ways of knowing. That [is] balanced within a system and organization that is fundamentally focused on individuals and illness. We are doing our best... 

Decreases in CDP activities were more prevalent among FMOs than NGOs (Table5). More than 50% of FMOs reported that activities targeting physical activity, healthy eating, healthy lifestyle and healthy weights had either decreased or been suspended. Less than 20% of NGOs reported such decreases. Higher proportions of NGOs than of FMOs reported increases in CDP activities.

**Table 5:** Percentage of user FMOs and NGOs undertaking specific CDP activities, according to the status of each activity during the COVID-19 pandemic, PHORCAST, Canada, 2023 (n = 169)

Area	FMO					NGO				
	Organizations that offered activity in past 3 years, n	Suspended/ discontinued, %	Decreased, %	Remained stable, %	Increased, %	Organizations that offered activity in past 3 years, n	Suspended/ discontinued, %	Decreased, %	Remained stable, %	Increased, %
Lifestyle risk factors										
Tobacco control	70	26	23	40	11	29	10	7	66	17
Healthy eating	67	31	21	39	9	57	9	5	68	18
Physical activity	67	39	16	40	4	75	4	8	69	19
Healthy lifestyle	64	31	20	38	11	72	4	6	74	17
High blood pressure	20	20	10	55	15	18	11	6	61	22
Elevated cholesterol	15	13	7	67	13	10	0	10	60	30
Chronic disease diagnoses										
COPD	23	22	22	48	9	22	5	9	59	27
Diabetes	29	24	14	48	14	26	8	8	65	19
Cancer	28	14	18	43	25	26	12	4	54	31
Heart disease	24	21	21	54	4	23	9	4	74	13
Healthy weights	47	34	19	40	6	38	5	13	71	11
Mental health										
Stress	52	23	25	29	23	52	4	4	56	37
Mental health	69	19	22	22	38	65	2	5	54	40
Population groups										
Marginalized populations	75	12	26	31	31	67	3	6	57	34
Racialized groups/communities	67	13	24	36	27	51	4	6	49	41
Specific gender groups	55	15	20	40	25	39	3	5	51	41
Rural communities	72	15	26	44	14	60	3	8	65	23
Urban communities	63	14	29	44	13	57	4	7	70	19

**Abbreviations: **CDP, chronic disease prevention; COPD, chronic obstructive pulmonary disease; FMO, formally mandated organization; NGO, non-governmental organization. 

***Perception of pandemic-related restrictions as a barrier to or facilitator of CDP activities***

**Figure 1:**
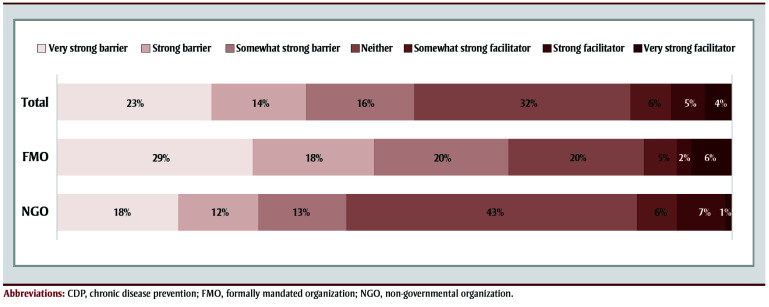
Percentage of user organizations overall and FMOs and NGOs, by perception of pandemic-related public health contagion measures as a barrier or facilitator to CDP activities, PHORCAST, Canada, 2023

Half (53%) of user organizations overall (67% of user FMOs and 43% of user NGOs) perceived the public health measures to stop the spread of COVID-19 as a barrier to CDP activities (Figure 1). One-third (32%) reported that the public health measures were neither a facilitator nor a barrier (20% of NGOs and 43% of FMOs). Of note, 15% overall viewed the measures as a facilitator, and this view was consistent across FMOs and NGOs (13% and 14%).

The following participant comment exemplifies how the COVID-19 pandemic served as a barrier to sustaining CDP activities by diverting staff and resources away from established CDP efforts:

As a smaller public health unit, nearly all [our] resources were deployed to the COVID-19 pandemic response. Currently, we are in the recovery phase and are in the process of planning and prioritization, within a new organizational structure. At this time [2023], we have not resumed most CDP activities. Prior to the COVID-19 pandemic, we had a dedicated CDP team which focused on multilevel activities. We hope to get back to this level of service delivery. 

## Discussion

In this study, our aim was to describe the impact of the COVID-19 pandemic on the resources and activities of public health organizations across Canada with mandates for CDP. A sizable proportion of these public health organizations reported major or minor pandemic-related decreases in CDP funding, personnel and activities. Changes were generally similar across resource and user NGOs, but were more pronounced among FMOs, and especially user FMOs. Relatively high proportions of organizations reported reductions in tobacco control, healthy eating, physical activity and healthy weights activities; activities for mental health and stress and targeting underserved groups (i.e. marginalized populations, racialized groups and specific gender groups) increased. Further, more than half of user organizations perceived the public health measures implemented to restrict the spread of COVID-19 as a barrier to CDP activities.

Although re-allocation of resources during public health emergencies may be inevitable, there should be widespread recognition across public health and health services jurisdictions that the burden of chronic disease will be affected by these re-allocations.^14^-^16^ Individuals with chronic diseases^17^-^20^ and those with risk factors for chronic disease (i.e. tobacco use, unhealthy diets, physical inactivity)^21^-^23^ were more vulnerable to severe COVID-19 outcomes and increased mortality. Reinforcing CDP capacity should be considered a key component of pandemic preparedness and response.

Fewer resource organizations than user organizations reported pandemic-related changes. This could be because resource organizations do not rely as heavily on in-person interactions in their day-to-day activities, which would have facilitated operational continuity during lockdowns and when physical distancing measures were in place. These organizations may have been able to shift more easily to online and digital platforms. A 2020 systematic review highlights the limited evidence for the effectiveness of mobile health (or mHealth) interventions and tools (e.g. mobile apps, text messaging) in managing conditions such as diabetes and obesity.^24^ Research on digital interventions in weight management and healthy lifestyle behaviours emphasizes the importance of behavioural theories, user-centred design, personalization, timely feedback and motivation, addressing access barriers and collaboration between developers, health care professionals and users.^25^ However, further study is needed to assess the feasibility and impact of digital strategies in the Canadian public health context.

The most pronounced decreases in CDP resources and activities occurred among user FMOs. While NGOs showed some stability and even increases in certain areas, FMOs more often reported decreases. This difference likely reflects the inherent organizational structures and funding mechanisms that distinguish these types of organizations. Namely, inflexibility in FMO processes, structures or practices may affect their ability to adapt quickly to crises or natural disasters without significant bureaucratic changes.^26^ In contrast, NGOs might have more flexible operational structures that allow them to create interdepartmental task forces and rapidly revise emergency response protocols, and diversified funding sources, such as private donations and grants,^27^ which may better position them to maintain or swiftly adapt their services and continue their engagement with underserved populations. It is also worth noting that user FMOs are often staffed with individuals who operate under a dual mandate to address both infectious and chronic (or noncommunicable) diseases, which may have resulted in staff transfers from CDP to infectious disease programs during crises.

Despite these challenges, both FMOs and NGOs demonstrated similar activity levels in CDP domains over the past 3 years, with FMOs more active in the areas of lifestyle risk factors, such as tobacco control and healthy eating, and addressing the CDP needs of marginalized and racialized populations. However, decreases in and suspensions of CDP activities were reported across all types of activities, reflecting the widespread impact of the pandemic. Compared to NGOs, FMOs generally experienced more decreases and suspensions, suggesting the need for tailored strategies and pre-pandemic planning to help FMOs maintain key CDP activities during public health crises. Future research should explore the potential for enhanced FMO–NGO collaboration^28^ during crises. Understanding how these organizations might synergize resources and activities may provide actionable strategies to foster resilient public health responses. Moeenian et al.^29^ found that factors such as investing in NGO collaboration, management ability and cultural and educational infrastructure are critical to the success of such initiatives. Exploring these collaborative strategies in Canada could provide valuable insights that help foster resilient public health responses.

We drew measures of CDP resources and activities from an empirically supported integrative conceptual model of organizational capacity for CDP.^12^ This model identifies several critical elements—organizational capacity, determinants, facilitators, outcomes and the broader social determinants of health—that are thought to influence the effectiveness of CDP activities. According to this model, resources, skills and infrastructure are essential for effective CDP efforts. However, our observations suggest depletions in resources during the pandemic. While not measured directly in this study, organizational determinants such as commitment, technical expertise and leadership may have also been strained due to priorities shifting toward urgent pandemic responses. In addition, changes in facilitators such as governmental and public priorities, which are considered mediators between organizational capacity and outcomes,^12^ might have influenced the level of engagement in CDP activities. Further research could be valuable in quantifying these impacts and exploring strategies to maintain organizational capacity during such shifts.

***Limitations***

Limitations of this study include that data were collected from one participant within each organization, although each of these individuals was carefully selected as the most knowledgeable about CDP. Organizational characteristics should ideally be assessed using objective measures (e.g. data from health records, registries or databases that track implementation of CDP activities) to the extent possible. Self-report data are subject to misclassification error. However, because of feasibility and cost, self-report is the most common data collection method in organizational research.^30^

## Conclusion

This work offers novel insight into changes in CDP resources and activities during the COVID-19 pandemic that may affect the burden of chronic disease in Canada. We documented important declines in funding, personnel and CDP activities across public health organizations. Although efforts targeting mental health and underserved populations increased, many traditional CDP activities were suspended. These findings underscore the necessity for building and maintaining resilient public health systems capable of sustaining prioritization of CDP efforts during public health crises. Continued monitoring of CDP resources and activities is essential to ensure that it remains a top public health priority. Using the lessons learned from the early years of the COVID-19 pandemic, it is essential to prioritize and rebuild CDP infrastructure to ensure that public health systems are resilient and capable of addressing both ongoing and future health challenges effectively.

## Acknowledgements

The authors thank Martha Ta, Teodora Riglea and Jodi Kalubi for their contributions to data collection, management and/or analyses. Katerina Maximova holds Murphy Family Foundation Chair in Early Life Interventions. Jennifer O’Loughlin held a Tier 1 Canada Research Chair in the Early Determinants of Adult Chronic Disease 2006-21. Maryam Marashi holds a Social Sciences and Humanities Research Council of Canada (SSHRC) doctoral fellowship.

## Funding

This study was supported by operational funds from the Canadian Institutes of Health Research (grant #170321).

## Conflicts of interest

Jennifer O’Loughlin is one of this journal’s Editorial Board Members, but was not involved in the editorial decision-making associated with this manuscript.

The authors have no competing interests.

## Authors’ contributions and statement

KM: Supervision, conceptualization, funding acquisition, project administration, resources, writing – review and editing.

MM: Analysis, writing – original draft, writing – review and editing.

EH: Conceptualization, writing – review and editing.

DM: Conceptualization, writing – review and editing.

GP: Conceptualization, writing – review and editing.

GP: Conceptualization, writing – review and editing.

JLM: Conceptualization, methodology, supervision, writing – review and editing.

The content and views expressed in this article are those of the authors and do not necessarily reflect those of the Government of Canada.
